# Supplementary material for: Effects of galvanic vestibular stimulation on bodily ownership and postural control: An experimental examination with counterbalanced randomization of stimulus conditions
Source: PLoS One. 2026 May 8;21(5):e0348060. doi: 10.1371/journal.pone.0348060 (PMC13155589; doi:10.1371/journal.pone.0348060)
Supplement: S1 Table — This table provides the Shapiro–Wilk test statistics and associated p-values for the RHI scores, SLS times, and FST angular deviations across all four conditions (Baseline, Sham, 30-min GVS, and 60-min GVS). (DOCX) [file pone.0348060.s001.docx]

**Supplementary**

Table S1. Shapiro–Wilk normality test results for outcome measures and change scores

| Variable | Condition | Shapiro–Wilk W | p-value | Distribution |
| --- | --- | --- | --- | --- |
| RHI | Baseline | 0.956 | 0.067 | Normal |
|  | Sham | 0.951 | 0.044 | Non-normal |
|  | 30 min | 0.963 | 0.129 | Normal |
|  | 60 min | 0.909 | 0.001 | Non-normal |
| SLS – Left | Baseline | 0.948 | 0.033 | Non-normal |
|  | Sham | 0.968 | 0.205 | Normal |
|  | 30 min | 0.969 | 0.237 | Normal |
|  | 60 min | 0.921 | 0.003 | Non-normal |
| SLS – Right | Baseline | 0.935 | 0.011 | Non-normal |
|  | Sham | 0.954 | 0.060 | Normal |
|  | 30 min | 0.936 | 0.011 | Non-normal |
|  | 60 min | 0.974 | 0.362 | Normal |
| FST | Baseline | 0.852 | <0.001 | Non-normal |
|  | Sham | 0.948 | 0.034 | Non-normal |
|  | 30 min | 0.970 | 0.261 | Normal |
|  | 60 min | 0.937 | 0.013 | Non-normal |
| ΔRHI | 30 − Baseline | 0.968 | 0.236 | Normal |
|  | 60 − Baseline | 0.959 | 0.112 | Normal |
|  | Sham − Baseline | 0.970 | 0.290 | Normal |
| ΔSLS – Left | 30 − Baseline | 0.885 | <0.001 | Non-normal |
|  | 60 − Baseline | 0.871 | <0.001 | Non-normal |
|  | Sham − Baseline | 0.884 | <0.001 | Non-normal |
| ΔSLS – Right | 30 − Baseline | 0.867 | <0.001 | Non-normal |
|  | 60 − Baseline | 0.796 | <0.001 | Non-normal |
|  | Sham − Baseline | 0.891 | <0.001 | Non-normal |
| ΔFST | 30 − Baseline | 0.852 | <0.001 | Non-normal |
|  | 60 − Baseline | 0.938 | 0.018 | Non-normal |
|  | Sham − Baseline | 0.939 | 0.020 | Non-normal |
