# Supplementary material for: Effects of galvanic vestibular stimulation on bodily ownership and postural control: An experimental examination with counterbalanced randomization of stimulus conditions
Source: PLoS One. 2026 May 8;21(5):e0348060. doi: 10.1371/journal.pone.0348060 (PMC13155589; doi:10.1371/journal.pone.0348060)
Supplement: Attachment — Submitted filename: Response_to_Reviewers 27.02.2026.docx [file pone.0348060.s005.docx]

**Response to Reviewers**

Dear Editor,

Thank you for the positive evaluation of our manuscript titled "Effects of Galvanic Vestibular Stimulation on Bodily Ownership and Postural Control: An Experimental Examination with Counterbalanced Randomization of Stimulus Conditions" (PONE-D-25-54398R1).

We appreciate the meticulous review and the specific correction provided regarding the references. We have addressed the point raised as follows:

**Reviewer #4 Comment:** Reference 25 (doi: 10.1111/j.1749-6632.2011.06269.x) lists the authors incorrectly.

**Response:** We apologize for this oversight. We have corrected the author names for this reference in the revised manuscript. The reference (now cited as Reference 22) correctly lists the authors.

We have also performed a final check of the entire reference list to ensure accuracy and compliance with PLOS ONE requirements.

Thank you for your guidance throughout this process.

Sincerely,

**Dr. Kerem Ersin**
